# RNA Sequencing Reveals Novel Transcripts from Sympathetic Stellate Ganglia During Cardiac Sympathetic Hyperactivity

**DOI:** 10.1038/s41598-018-26651-7

**Published:** 2018-06-05

**Authors:** Emma N. Bardsley, Harvey Davis, Olujimi A. Ajijola, Keith J. Buckler, Jeffrey L. Ardell, Kalyanam Shivkumar, David J. Paterson

**Affiliations:** 1 Wellcome Trust OXION Initiative in Ion Channels and Disease, Burdon Sanderson Cardiac Science Centre, Department of Physiology, Anatomy and Genetics, Sherrington Building, University of Oxford, Oxford, OX1 3PT UK; 2 UCLA Cardiac Arrhythmia Center, 100 Medical Plaza, Suite 660, Los Angeles, CA 90095 USA

## Abstract

Cardiovascular disease is the most prevalent age-related illness worldwide, causing approximately 15 million deaths every year. Hypertension is central in determining cardiovascular risk and is a strong predictive indicator of morbidity and mortality; however, there remains an unmet clinical need for disease-modifying and prophylactic interventions. Enhanced sympathetic activity is a well-established contributor to the pathophysiology of hypertension, however the cellular and molecular changes that increase sympathetic neurotransmission are not known. The aim of this study was to identify key changes in the transcriptome in normotensive and spontaneously hypertensive rats. We validated 15 of our top-scoring genes using *q*RT-PCR, and network and enrichment analyses suggest that glutamatergic signalling plays a key role in modulating Ca^2+^ balance within these ganglia. Additionally, phosphodiesterase activity was found to be altered in stellates obtained from the hypertensive rat, suggesting that impaired cyclic nucleotide signalling may contribute to disturbed Ca^2+^ homeostasis and sympathetic hyperactivity in hypertension. We have also confirmed the presence of these transcripts in human donor stellate samples, suggesting that key genes coupled to neurotransmission are conserved. The data described here may provide novel targets for future interventions aimed at treating sympathetic hyperactivity associated with cardiovascular disease and other dysautonomias.

## Introduction

Sympathetic hyperactivity is a key feature of human hypertension that is also seen in animal models of cardiovascular disease^1,2^. In the spontaneously hypertensive rat (SHR), a genetic-based model of hypertension, dysautonomia occurs prior to increases in arterial blood pressure^3–5^. This neural phenotype is a powerful predictor of morbidity and mortality^6–8^. Indeed, vagal nerve stimulation^9,10^ and sympathetic stellectomy^11^ have had some success in the prevention of arrhythmia where neuromodulation is now being considered as an adjunct therapy^12^.

The sympathetic stellate ganglia (cervicothoracic ganglia) are located alongside vertebrate T1 to T3. They are the primary sympathetic ganglia that innervate the heart and as such, exert the greatest control over increases in heart rate and contractility. The precise mechanisms that underpin enhanced sympathetic nerve activity (SNA) in disease are unknown, however, research indicates that high intracellular Ca^2+^ ([Ca^2+^]_i_)^2,4,13^ and impaired Ca^2+^ handling linked to oxidative stress^14,15^ are key features in this process and predispose to dysautonomia^2,4,13,16^.

In the spontaneously hypertensive rat (SHR), postganglionic sympathetic neurons (PGSNs) display increased [Ca^2+^]_i_ transients as a result of increased *N*-type voltage-gated Ca^2+^ (Ica_N_, Ca_v_2.2) channel activity^2,17^ and impaired endoplasmic reticulum (ER) and mitochondrial Ca^2+^ handling^4^. Cyclic nucleotides (cNs) and their effectors regulate Ca^2+^ conductance predominantly via phosphorylation of ion channels, however, the relationship between cN second messenger signalling pathways and abnormal [Ca^2+^]_i_ is still poorly understood in disease^2,3^. Moreover, specific gene ontologies and protein-protein interactions linked to abnormal Ca^2+^ regulation in sympathetic neurons from hypertensive states is lacking.

Here, we carried out an RNA sequencing transcriptomic analysis to identify differential gene expression in sympathetic neurons from normotensive and hypertensive rats. Importantly, our RNAseq results demonstrated significant differential expression in transcripts that regulate [Ca^2+^]_i_ signalling in addition to genes that regulate cN signalling in disease. We validated our findings using quantitative real-time polymerase chain reaction (*q*RT-PCR) and subsequently assessed whether these genes were present in sympathetic stellate ganglia from human donors, in order to provide a framework for future validation and targeting.

## Results

### Study Design

**Figure 1:**
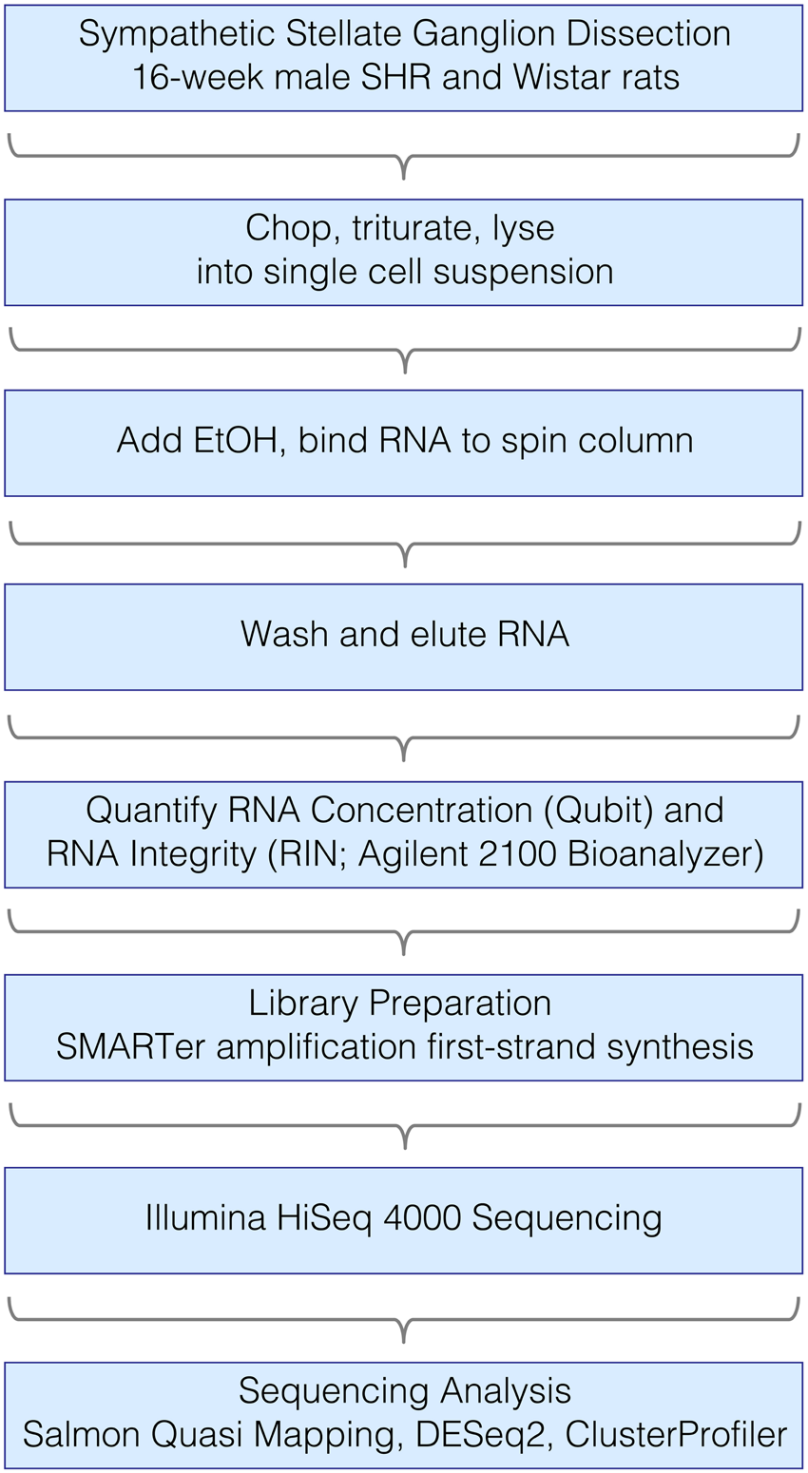
RNA Sequencing Pipeline.

To study the rat stellate ganglia transcriptome, we obtained RNA from four male 16-week-old SHR with hypertension and four male age-matched Wistar control rats. RNA extracts from these ganglia contained ~8.5 ng/μL RNA. RNA integrity (RIN) was validated using an Agilent 2100 Bioanalyzer. To ensure good quality RNA we only used samples with RINs >8.5 for sequencing. Using the HiSeq 4000 sequencing platform, we generated ~345 million reads of 75-bp paired-end RNAseq data, i.e. ~22 million reads per sample. We performed a comparative analysis of the stellate ganglia transcriptome between Wistar and SHR phenotypes. The number of animals per group (n = 4) and the sequencing parameters established were based on the recommendations of the Wellcome Trust Centre for Human Genomics, in addition to those published by Conesa *et al*.^18^. Figure 1 illustrates our RNAseq pipeline.

### Quasi-mapping and Differential Expression Analysis

**Figure 2:**
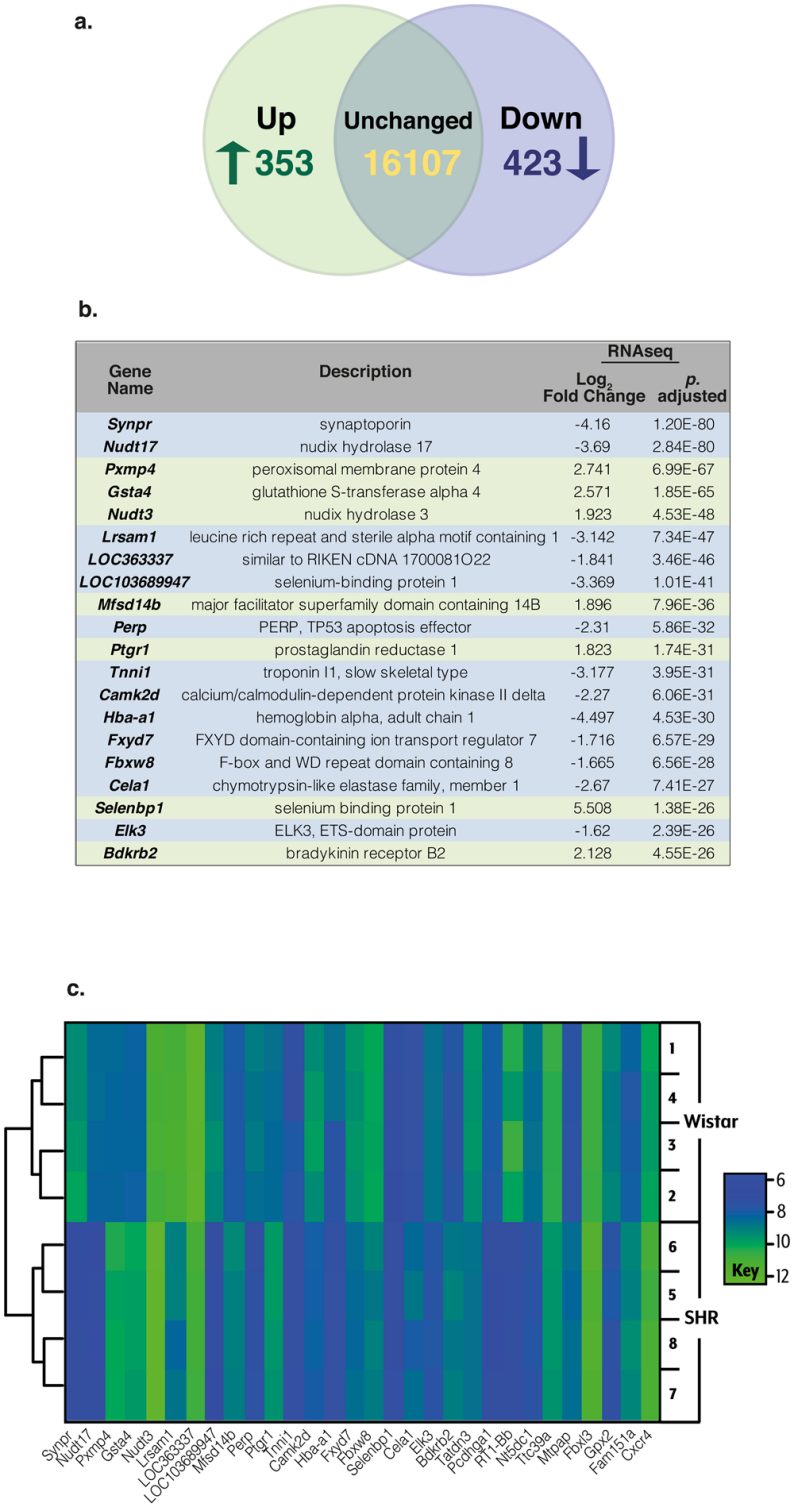
Overview of the differentially expressed genes in the stellate ganglia. The Venn diagram represents the total number of upregulated gene transcripts (353) and downregulated transcripts (423) in the SHR stellate ganglia, identified by DESeq2 analysis (**a**). The top differentially expressed transcriptomic changes between Wistar and SHR sympathetic stellate ganglia are listed; where green and blue represent up- and downregulated genes respectively (**b**). The heat-map represents the expression levels of differentially expressed genes in Wistar and SHR stellate ganglia. The colour legend indicates similarity of gene expression between samples. The colour gradients of samples indicate the inter-sample similarity of transcript expression for a particular gene. The dendrogram depicts overall similarity of the samples within this geneset, highlighting the distinction between strains as well as between samples within strains (**c**).

Transcript level quantification was performed using the quasi-mapping function of the Salmon package, where inbuilt functions corrected for GC and positional bias^19^. These data were converted into quantitative gene level expression values, and the differential gene expression between the strains was calculated and normalised using DESeq2^20^. We obtained an average number of mapped reads (74.75%) that was not significantly different between groups (Table 1). DESeq2 was used to generate an MA plot for visual representation of our genomic data (Supplementary Fig. 1a) and a Principal Component Analysis (PCA) plot with log normalisation was produced to demonstrate variation within our samples (Supplementary Fig. 1b)^20^. As confirmation of the expected neuronal phenotype, we identified that markers consistent with sympathetic neurons were among the most highly expressed genes in both strains (e.g. Dopamine-β hydroxylase, *Dbh;* and Neuropeptide Y, *Npy*; see Table 2). The differential expression analysis identified 776 differentially expressed genes in the stellate ganglia, where approximately 55% of genes were upregulated and 45% downregulated in the SHR. The top 20 differentially expressed genes are displayed (Fig. 2).

**Table 1:** Read mapping rates via Salmon’s quasi-mapping function.

Sample	Strain	Mapping rate	Number of mapped reads
Sample1	Wistar	78.66%	35509076
Sample2	Wistar	75.57%	30112270
Sample3	Wistar	78.38%	34634784
Sample4	Wistar	75.18%	33764576
Sample5	SHR	70.63%	31194868
Sample6	SHR	78.47%	25820298
Sample7	SHR	68.50%	32573670
Sample8	SHR	72.60%	34026720

Mapping rate indicates the percentage of assigned reads relative to the total number of reads. The numerical value for mapped reads per sample are also shown.

**Table 2:** Defining the transcriptome of Wistar and SHR stellate ganglia.

Wistar		SHR	
Gene	Mean Counts	Gene	Mean Counts
*Tuba1a*	877116.071	*Tuba1a*	922096.7557
*Ubb*	616451.3926	*Rn28s*	675041.3777
*Rn28s*	531551.2116	*Ubb*	583166.9017
*Tubb3*	489766.6486	*Tubb3*	489906.3397
***Dbh***	365271.5768	*Rn18s*	307588.9061
*Rn18s*	314117.4054	***Dbh***	299093.8225
*Aldoa*	260208.4448	*Hspa8*	245512.6941
*Thy1*	251700.8758	*Actg1*	241889.488
*Ndrg4*	248263.1557	*Aldoa*	236588.5852
*Hspa8*	245297.6199	*Ndrg4*	224655.0884
*Actg1*	241090.67	*Actb*	195887.7534
*Actb*	227909.538	*Thy1*	192753.2441
*Ubc*	167733.1003	***Npy***	192441.4061
***Npy***	166746.7384	*Hsp90ab1*	167854.4376
*Fth1*	166495.3895	*LOC310926*	167425.7802
*Tuba1b*	166011.3638	*Gapdh*	166721.7809
*Gapdh*	164910.0889	*Tuba1b*	165999.6912
*Uchl1*	153108.9558	*Uchl1*	137628.8969
*Hsp90ab1*	151214.2007	*Stmn2*	134260.5427
*Stmn2*	139862.0444	*Pkm*	129310.0511
*LOC310926*	132381.9518	*Ubc*	128414.6292
*Zwint*	125736.4614	*Cyb561*	124946.1778
*Pkm*	122043.8767	*Fth1*	124938.6603
*Cyb561*	114908.2288	*Eno1*	121470.1003
*Eno1*	114075.9204	*Fxyd6*	120968.7869
***Snap25***	112163.485	*Ppia*	113541.3151
*Eef1a1*	111814.9377	*Tubb5*	112056.0616
*Tubb5*	111260.3167	*Sncg*	110216.3007
*Sncg*	109948.206	*Zwint*	109996.2104
***Th***	105911.6304	*Eef1a1*	109106.2249

The table includes a list of the top 30 genes and their respective mean counts in stellate ganglia obtained from Wistar and SHR. Genetic markers of sympathetic neurons are highlighted in bold. No significant difference was found for any of the sympathetic or neuronal markers in this list.

### Gene Ontology Analysis

**Figure 3:**
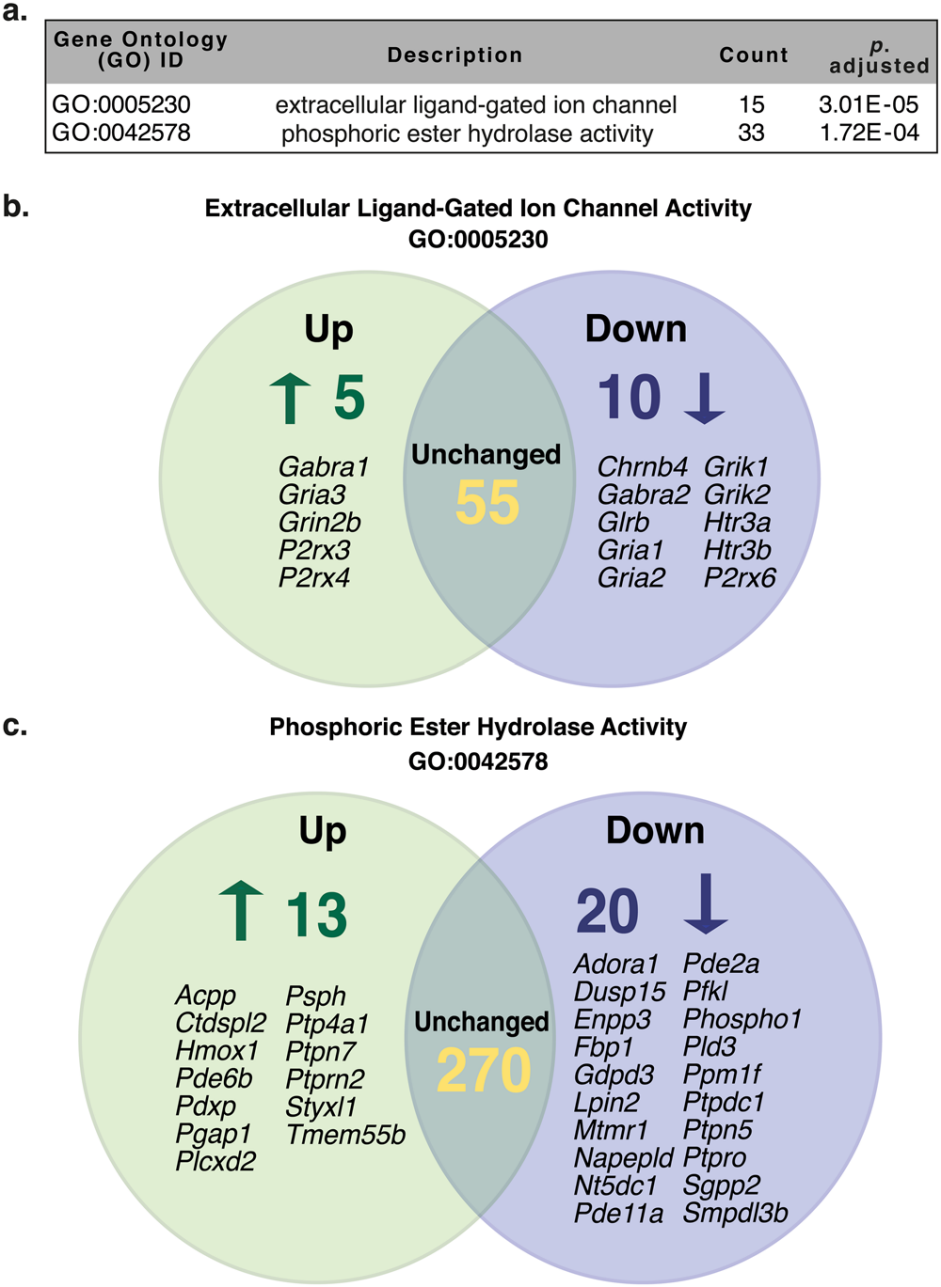
Gene Ontology: Molecular Function Category. The top two over-represented molecular function (MF) gene ontology (GO) groups include ‘extracellular ligand-gated ion channel activity’ (GO:0005230) and ‘phosphoric ester hydrolase activity’ (GO:0042578; **a**). Venn diagrams depict the upregulated genes (green) and downregulated genes (blue) in each GO group (**b**,**c**; respectively). All other over-represented GO groups are included in the supplementary dataset.

To assess the relevance of the observed differentially expressed genes, we grouped these genes into Gene Ontology (GO) families^21^ using the ClusterProfiler R package^22^. We identified significant differences between the SHR and Wistar transcriptome in the following GO categories: Molecular Function (MF) and Cellular Component (CC). Semantically redundant groups were removed using the ClusterProfiler R package^22^. For the purpose of this study, we aimed to assess the differences in the SHR PGSN transcriptome that may be linked to the observed sympathetic hyperactivity and enhanced neurotransmission; therefore, we chose to focus our attention on the over-represented MF GO groups (at *p*.adj < 0.01). The most highly significant GO group within the MF category was identified as ‘extracellular ligand-gated ion channel activity’ (Fig. 3; GO:0005230; *p*.adj = 3 × 10^−5^). The other significantly over-represented GO groups in the SHR stellate ganglion include (in order of significance): ‘phosphoric ester hydrolase activity’ (Fig. 3; GO:0042578; *p*.adj = 1.72 × 10^−4^), ‘organic acid binding’ (Supplementary Table 1; GO:0043177; *p*.adj = 2.90 × 10^−3^), ‘glutamate receptor activity’ (Supplementary Table 1; GO:0008066; *p*.adj = 2.90 × 10^−3^), ‘gated channel activity’ (Supplementary Table 1; GO:0022836; *p*.adj = 3.59 × 10^−3^) and ‘carboxylic acid binding’ (Supplementary Table 1; GO:0031406; *p*.adj = 4.93 × 10^−3^). Of the over-represented MF GO groups, we found significant overlap between the genes identified in ‘extracellular ligand-gated ion channel activity’ (GO:0005230) ‘glutamate receptor activity’ (GO:0008066) and ‘gated channel activity’ (GO:0022836) as these GO groups form sub-categories of the parent GO group ‘ligand-gated ion channel activity’ (GO:0015276). In addition, ‘organic acid binding’ (GO:0043177) and ‘carboxylic acid binding’ (GO:0031406) are sub-branches of the larger parent group ‘small molecule binding’ (GO:0036094). Full lists of the significantly different MF and CC GO groups identified in SHR PGSNs are included in the supplementary data (Supplementary Tables 1, 2). Importantly, the differentially expressed transcripts within these GO groups may be linked to sympathetic dysfunction observed in the SHR model. There were no significantly different GO groups in the Biological Process (BP) category between Wistar and SHR.

### Differential Expression of Extracellular Ligand-Gated Ion Channel Genes: Confirmation by qRT-PCR

**Figure 4:**
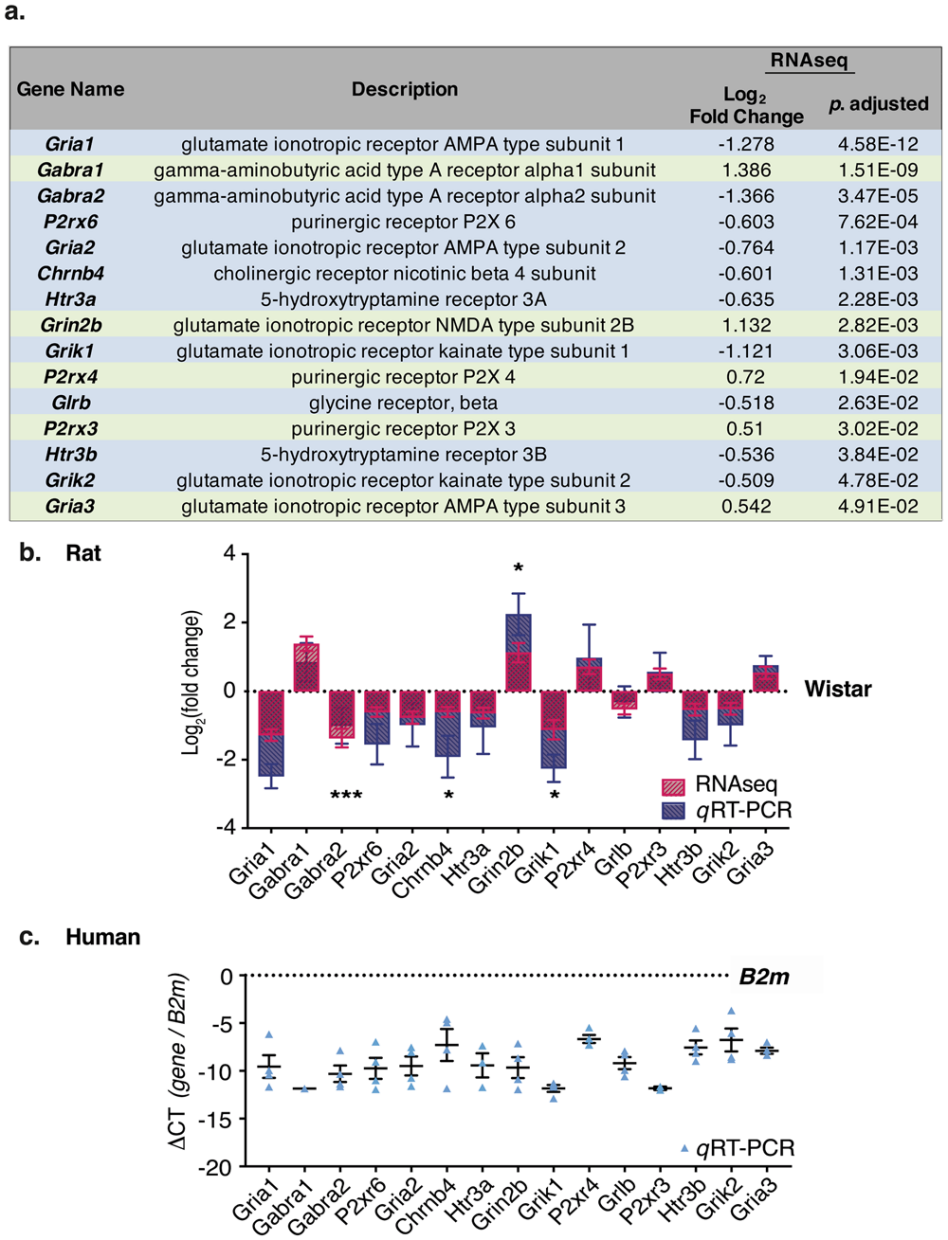
Extracellular Ligand-Gated Ion Channel Activity GO Group (GO:0005230). The differentially expressed genes in the MF GO group ‘extracellular ligand-gated ion channel activity’ (GO:0005230) are listed, where green and blue represent up- and downregulated genes respectively (**a**). The presence of these genes was validated using *q*RT-PCR and the ΔΔCT comparative method was applied for analysis (SHR/Wistar); with β2 microglobulin *(B2m)* selected as the housekeeping gene for comparison as there was no difference in *B2m* expression between strains in the RNAseq dataset. Log_2_ fold change of the differentially expressed genes in SHR stellates as identified by RNAseq are represented in red (**b**). Log_2_ fold change ± SEM of *q*RT-PCR SHR data are also depicted (blue; **b**) relative to Wistar. Significance is indicated above the relevant bars. The RNAseq trends for up- or downregulation of each gene was confirmed with *q*RT-PCR; however, only *Chrnb4 (p* < 0.05*)*, *Gria1 (p* < 0.001*)*, *Grik1 (p* < 0.05*)* and *Grin2b (p* < 0.05*)* were significantly different to the expression levels measured in Wistar samples. The presence of these genes was confirmed in human stellate ganglia samples (**c**). To quantify mRNA expression in human stellate samples, data were normalised to the housekeeping gene *B2m* and the ΔCT method was applied for analysis.

We obtained four independent Wistar and SHR RNA stellate ganglia to investigate whether we could replicate the RNAseq differential gene expression using *q*RT-PCR. In the 15 selected genes, we obtained similar trends using *q*RT-PCR as that observed in the RNAseq data; although due to variation within samples, only 4:15 genes reached significance (Fig. 4, unpaired two-tailed students *t*-test; Wistar vs. SHR). These included *Chrnb4* (Cholinergic Receptor Nicotinic β4 Subunit), *Gria1* (Glutamate Ionotropic Receptor AMPA Type Subunit 1), *Grik1* (Glutamate Ionotropic Receptor Kainate Type Subunit 1) and *Grin2b* (Glutamate Ionotropic Receptor NMDA Type Subunit 2B).

### Extracellular Ligand-Gated Ion Channel Genes Are Expressed in Human Sympathetic Stellate Ganglia

To assess whether these genes are conserved in other species and have potential relevance for human sympathetic activity, we validated the expression of these genes in ganglia obtained from human donors. Given the limited availability of human tissue from patients, it was not possible to assess the role of these genes in human disease itself. Importantly, however, we detected the presence of glutamate (AMPA, NMDA and Kainate) and GABA receptor subunits which have not been previously identified within the human sympathetic stellate ganglia (Fig. 4). We also identified the presence of purinergic, glycinergic and serotonergic receptors.

### Differential Expression of Phosphoric Ester Hydrolase Activity Genes

**Figure 5:**
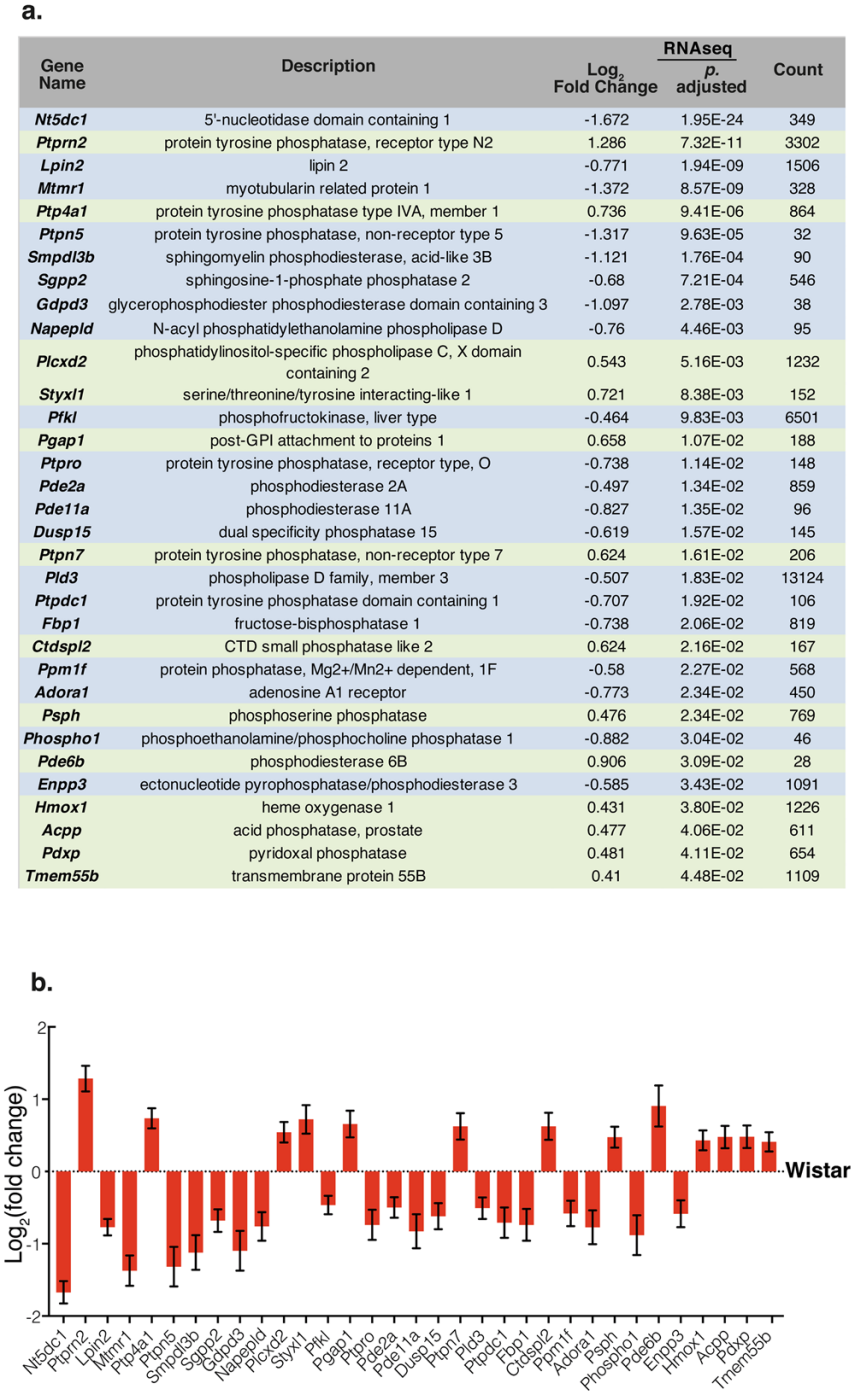
Phosphoric Ester Hydrolase Activity GO Group (GO:0042578). The differentially expressed genes in the MF GO group ‘phosphoric ester hydrolase activity’ (GO:0042578) are listed, where green and blue represent up- and downregulated genes respectively (**a**). Log_2_ fold change of the differentially expressed genes of interest in SHR relative to Wistar as identified by RNAseq (**b**).

The ‘phosphoric ester hydrolase activity’ (GO:0042578) GO group was found to contain the highest number of differentially expressed genes in the MF category. We identified 13 upregulated genes including *Pde6b* and 20 downregulated mRNA transcripts including *Pde11a* and *Pde2a* (Figs 3 and 5). The expression of 270 genes in this group was unchanged. Within this family, we identified that most of the affected transcripts were those encoding phosphatase enzymes.

### KEGG Functional Enrichment Pathways are Impaired in SHR Stellate Ganglion

**Figure 6:**
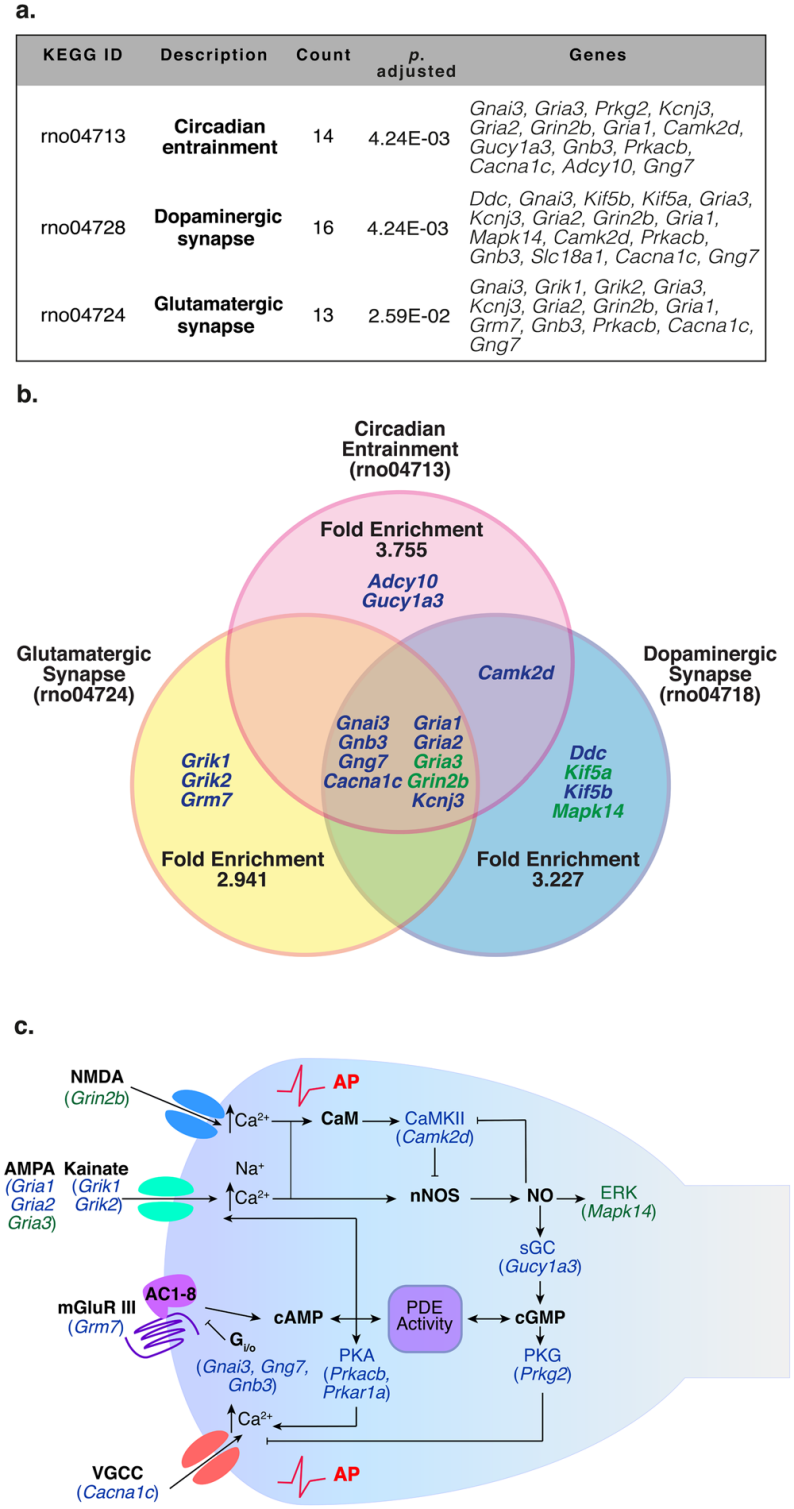
KEGG Enrichment Pathways in SHR versus Wistar Stellate Ganglion. We identified 3 KEGG functional enrichment pathways that are particularly relevant to post-synaptic postganglionic signalling (**a**). The highest over-represented group was ‘Circadian Entrainment’ (rno04713; *p*. adj = 4.24 × 10^−3^) followed by ‘Dopaminergic Synapse’ (rno04728; *p*. adj = 4.24 × 10^−3^) and ‘Glutamatergic Synapse’ (rno04724; *p*. adj = 2.59 × 10^−2^). The Venn Diagram depicts the upregulated genes (green) and downregulated genes (blue) and highlights the overlap between each of the respective over-represented KEGG groups (**b**). Other over-represented KEGG groups are included in the supplementary data. In (**c**), we show an integrated KEGG enrichment diagram, combining signalling pathways from the over-represented KEGG pathways ‘Circadian entrainment’, ‘Dopaminergic synapse’ and ‘Glutamatergic synapse’. Here we highlight the possible involvement of these transcriptomic changes on intracellular signalling. This figure is primarily based on the supplied rno04713 KEGG diagram.

Using the ClusterProfiler R package^22^, we performed a Kyoto Encyclopedia of Genes and Genome (KEGG)^23–25^ pathway enrichment analysis, that links the observed differences within the SHR PGSN transcriptome to relevant intracellular signalling pathways. We analysed the relevance of the differentially expressed genes by performing a functional enrichment KEGG analysis that identified ‘Circadian Entrainment’ (rno04713; *p*.*adj* = 0.004); ‘Dopaminergic Synapse’ (rno04728; *p*.*adj* = 0.004); ‘Retrograde Endocannabinoid Signalling’ rno04723; *p*.*adj* = 0.0196); ‘Glutamatergic Synapse’ (rno04724; *p*.*adj* = 0.0259) and ‘Neuroactive Ligand-receptor Interaction’ (rno04724; *p*.*adj* = 0.0259) as the most over-represented pathways in the hypertensive strain (Fig. 6, Supplementary Table 3).

Importantly, many of the same genes were represented in each of these groups, therefore we combined the KEGG signalling pathways (Circadian Entrainment, Dopaminergic Synapse and Glutamatergic Synapse) to identify the possible involvement of these transcriptomic changes on intracellular signalling (Fig. 6b,c). The KEGG enrichment pathway observations indicate that RNA transcripts associated with glutamate signalling are differentially expressed within SHR cardiac PGSNs. Within these KEGG groups, genes associated with glutamate signalling are coupled to [Ca^2+^]_i_ directly, where differences in subunit composition of glutamate-activated ion channels affect Ca^2+^ permeability. Moreover, cyclic nucleotide generation and activation of their effectors are thought to influence the activity of these channels (Fig. 6b,c). The interaction between glutamate signalling and cyclic nucleotides within the peripheral nervous system has not been previously described.

## Discussion

We have used RNAseq to identify alterations in the transcriptome of the stellate ganglion between SHR and Wistar rats, in order to establish lead genetic candidates that may be linked to sympathetic hyperactivity in this hypertensive model^26–30^.

Here we report several novel findings. First, we observed differential expression in the GO group encoding extracellular ligand-gated ion channels that are linked to intracellular Ca^2+^ regulation. These changes were confirmed by *q*RT-PCR. Secondly, we provide evidence for the impaired expression for a family of genes that encode PDEs and phosphatase activity in hypertension. Altered transcription of PDEs and associated phosphatases that regulate PDE activity may be coupled to the dysregulation of cyclic nucleotide signalling, which is impaired in neurogenic hypertension^2^. Finally, to provide physiological context, we show that key differentially expressed genes are conserved and present in human stellate ganglia.

We observed high levels of transcripts involved in pathways governing adrenergic synthesis (e.g. *Dbh*, *Th*), neurotransmission (e.g. *Snap25*) and reuptake (e.g. *Slc6a2)*, corroborating similar recently published profiles of stellate neurons^31–33^. We found that the highest number of differentially expressed genes between Wistar and SHR stellate ganglia within the molecular function (MF) gene ontology (GO) category, were associated with the ‘extracellular ligand-gated ion channel’ (GO:0005230) and the ‘phosphoric ester hydrolase activity’ (GO:0042578) superfamilies. In particular, our data highlighted significant differences in the mRNA transcript expression levels of several glutamate-activated ion channel subunits including: upregulation of the NMDA receptor (R) NR2 subunit (*Grin2b*) and AMPA GluA3 subunits (*Gria3*); as well as downregulation of AMPA GluA1 and GluA2 subunits (*Gria1*, *2*) and Kainate 1 and 2 R subunits (*Grik1*, *2* respectively), supporting previous evidence for the role of amino acids in regulating cardiac-sympathetic activity^34–37^. Within the ‘extracellular ligand-gated ion channel’ GO Group, we also identified significant changes in the transcript expression levels of purinergic Rs (*P2rx3*, *4*, *6*) and GABA_A_ R subunits *(Gabra1*, *Gabra2)*. We also observed a significant downregulation of the ionotropic serotonin type 3 R subunits (*Htr3a*, *Htr3b*), nicotinic acetylcholine R β4 subunit (*Chrnb4)* and the chloride (Cl^−^)-permeable glycine channel β subunit (*Glrb*). Our RNAseq data provides an accurate prediction of the differential expression of these genes, as the trend for up- or downregulation was confirmed by *q*RT-PCR (Fig. 4). Moreover, using *q*RT-PCR we confirmed the presence of these genes within human male donor PGSNs (Fig. 4). Importantly, differential expression of ligand-gated ion channel subunits may have implications for ion permeability. The expression of these as-yet unreported ion channels on human sympathetic nerves has clear clinical relevance, as it may offer new targets for interventions aimed at modulating neurotransmission as well as considerations regarding the side effect profiles of current therapeutics.

We also analysed the relevance of differentially expressed genes by performing a functional enrichment KEGG analysis. This identified ‘Circadian Entrainment’, ‘Dopaminergic Synapse’, ‘Retrograde Endocannabinoid Signalling’, ‘Glutamatergic Synapse’ and ‘Neuroactive ligand-receptor Interaction’ as the most over-represented pathways in the hypertensive strain (Fig. 6). Within these KEGG enrichment pathways, the genes associated with glutamate signalling were the most heavily over-represented genes, supporting previous evidence that amino acids may play a role in regulating sympathetic activity in healthy physiological conditions^34,36,37^. The NMDAR NR2 subunit (*Grin2b*) that was found to be upregulated in SHR ganglia, is the binding site for glutamate and plays a direct role in regulating the electrophysiological properties of the channel^38–40^. Moreover, decreases in the AMPA subunit GluA2 (*Gria2*) mRNA expression may be associated with increased Ca^2+^ permeability. Indeed, in CA1 pyramidal neurons, increased cAMP and PKA signalling shift GluA2/3-containing AMPARs to a more conductive state^41,42^. Whether impaired cN signalling within SHR PGSNs affects AMPA R conductance and contributes to the observed Ca^2+^ dysfunction within sympathetic stellate neurons remains to be established.

Within the over-represented KEGG groups we identified several differentially expressed transcripts indicating that cGMP-PKG signalling may be downregulated. We have previously identified an impairment in cyclic nucleotide (cN) signalling linked to dysregulated [Ca^2+^]_i_ in hypertensive states^2,3^ and have demonstrated that enhancing cGMP signalling rectifies this feature^2^. We observed that the transcripts encoding the soluble guanylyl cyclase 1 soluble subunit α (*Gucy1a3*) and the cGMP-dependent Protein Kinase (PKG) type II subunit (*Prkg2*) are significantly downregulated in SHR ganglia. Importantly, we identified that the ‘phosphoric ester hydrolase activity’ (GO:0042578) was also significantly over-represented within the MF category. Within this GO group, we identified 33 significantly differentially expressed transcripts, including downregulation of *Pde11a* and *Pde2a* transcripts and upregulation of *Pde6b* (Fig. 5) supporting previous evidence that PDE dysregulation may play a role in cardiovascular diseases^13,16,43–46^. Recently, it was demonstrated that 1% of the Swedish population carry a loss-of-function mutation in PDE11a that is directly linked to hypertension, obesity and ischaemic stroke^47^. Moreover, a single nucleotide polymorphism (rs197163010) has been identified in PDE2a in the SHR/ola strain and reported in the NCBI dbSNP database^48^. Importantly, a number of other differentially expressed genes within this group also relates to the regulation of cell function through protein phosphorylation, including protein tyrosine and phosphoserine phosphatases that regulate protein kinase effector activity. A full overview of the differentially expressed transcripts are displayed in Fig. 5; however, it is pertinent to note that cN activity is dependent on PDE activity that is closely localised within specific subcellular microdomains^49^.

Several genes that we identified within these pathways have been previously associated with hypertension and CVDs. These include the class III metabotropic glutamate receptor 7 (*Grm7*)^50,51^, G-protein β3 subunit (*Gnb3*)^52–55^ and the voltage gated L-type Ca^2+^ Cannel α subunit (*Cacna1c*)^56,57^. Of particular relevance to this study is the finding that Protein Kinase A regulatory subunit 1a (*Prkar1a*) is downregulated in these cell types. *Prkar1a* transcription is integral for normal development of peripheral neurons including axonal sorting, myelination and proliferation of neurons^58^. Additional support for the importance of *Prkar1a* in peripheral neurons is found in patients with the inherited Carney Complex (CNC) syndrome^59,60^. Notably, patients with global loss-of-function mutations in *Prkar1a* develop peripheral nerve tumours and display a wide range of cardiovascular comorbidities that are often fatal, including dysregulated maintenance of blood pressure, cardiomyopathies and arrhythmia^60^. Indeed, in one study approximately 60% of CNC patients died of cardiovascular complications^59^.

### Limitations

In this study, we carried out a hypothesis neutral, non-biased approach to sequencing the transcriptome of sympathetic stellate neurons of SHR and normotensive Wistar rats. There are several limitations to this approach. First, the stellate ganglion comprises a heterogeneous population of cell types. This was apparent in the analysis, as we identified several markers of fibroblasts and astrocytes including vimentin and glial fibrillary acidic protein (GFAP) respectively, however; a high number of identified transcripts were of neuronal phenotype. Further studies to fully uncover the transcriptome of PGSNs require single cell RNAseq or confirmation of RNA transcripts in single PGSNs, using techniques such as *in situ* hybridisation for nucleic acids. Secondly, we used stellates obtained from male rats. While there is a plethora of data regarding the sex differences in hypertension and CVD incidence^61^, in this study we focussed on investigating the transcriptome of the male rat stellate ganglia, given that the prevalence for hypertension and CVD is significantly higher in males compared with premenopausal women^61^. Thirdly, given the low concentration of RNA in our stellate samples, we chose to amplify our samples during the library preparation using the SMARTer amplification protocol. We are aware that low level transcripts may be under-represented in the amplification process and in our dataset. Moreover, since we sequenced the transcriptome from rats with established hypertension, the transcriptomic changes that we identified may occur as compensatory rather than causative factors. Finally, our gene profiling and ontology analyses are enabled, yet limited, by the GO groups and KEGG enrichment pathways currently available.

Notwithstanding these limitations, RNAseq transcriptomics has revealed differential gene expression profiles in PGSNs obtained from SHR with established hypertension and normotensive Wistar rats. Leading transcripts were also present in human stellate ganglia. Taken together, these genes provide a starting framework to further investigate their role in sympathetic hyperactivity and other diseases associated with autonomic dysfunction.

## Methods

### Animals

16-week old male SHRs with established hypertension and age-matched normotensive Wistar control rats were obtained from Envigo, UK. At 16-weeks, it is well-established that SHR display hypertension and sympathetic hyperactivity^4,30,62–66^. In this study, we used the Wistar rat strain as the normotensive control, given that Wistar rats are the progenitor strain for WKY and the two strains display similar hemodynamic profiles at all ages as measured using multiple methods^4,66–72^. We have previously shown that neither Wistar nor WKY display a sympathetic Ca^2+^ phenotype (unlike the SHR)^13^ making the Wistar a suitable control in this study^73^. All rats were housed in standard plastic cages and artificial lighting was fixed to a natural 12-hour light/dark cycle. Food and water were available *ad libitum*. All experiments were performed in accordance with the UK Home Office Animal Scientific Procedures Act 1986 (ASPA) and approved by the University of Oxford (PPL 30/3131; David J. Paterson).

### RNA Extraction from Sympathetic Cardiac Ganglia

Right and left postganglionic sympathetic stellate neurons were dissected from 16-week-old male SHR and age-matched male Wistar rats. Right stellate ganglia were removed for RNA sequencing and the left stellate ganglia were used for matched *q*RT-PCR experiments. Rats were anaesthetised in an induction chamber (3–5% isoflurane) and humanely killed by a Home Office approved Schedule 1 method: overdose of pentobarbital (Euthatal, 200 mg/mL**)** and exsanguination. The stellate ganglia were placed in Hanks Buffered Saline Solution without Ca^2+^ and Mg^2+^. For clinical samples, human ganglia (n = 3 patients, two left and two right stellates) were extracted at UCLA and shipped on dry ice in RNA*later*® RNA Stabilization Solution (ThermoFisher). Rat and human ganglia were cleaned and de-sheathed and each sample contained RNA from one stellate. Briefly, stellate ganglia were transferred immediately to RLT lysis buffer (Qiagen) with β-mercaptoethanol (1%). Stellates were finely chopped and carefully triturated with fire-blown Pasteur pipettes until adequately digested. An equal volume of ethanol (70%) was added to each sample and vortexed. Rat RNA was extracted using an RNeasy Mini RNA Extraction Kit (Qiagen) and human RNA was extracted using an RNeasy Maxi RNA Extraction Kit (Qiagen) in accordance with the manufacturer’s instructions. RNA samples were divided and aliquoted for *q*RT-PCR and quality control experiments before snap freezing in liquid nitrogen and storage at −80 °C. The RNA quality and integrity from each sample, was confirmed using a 2100 Bioanalyzer Instrument with an RNA 6000 pico kit (Agilent). Rat samples with an RNA integrity number (RIN) less than 8.5 were discarded. Due to difficulties with fast shipping of human samples we accepted human RNA samples with RINs above 5.7. RNA concentrations were determined using a Qubit RNA High Sensitivity Assay Kit (Molecular Probes, Life Technologies) and a Qubit® 2.0 Fluorometer (Invitrogen, Life Technologies). Regarding the extraction of human ganglia; all methods were carried out in accordance with relevant guidelines and regulations. The human study was approved by an institutional committee, the UCLA Institutional review board, approval # 12-000701, and informed consent was obtained from all subjects.

### cDNA library preparation for RNA Sequencing

4 samples containing total RNA extracted from the right sympathetic stellate ganglion of 16-week-old male SHR (n = 4) and age-matched Wistar (n = 4) were sent to the High-Throughput Genomics Group at the Wellcome Trust Centre for Human Genetics (WTCHG) for RNAseq library construction and sequencing using an Illumina HiSeq 4000 (Illumina, Inc., San Diego, USA). The sequencing libraries were amplified using a SMARTer (first strand synthesis) amplification protocol, due to the low initial RNA concentrations obtained from a single stellate, and prepared for paired-end sequencing (2 × 75 bp). Each sample was sequenced on two separate lanes to minimise technical error and to increase the sequencing depth (~15–25 million reads per lane). Samples were randomized and blinded to the experimenter. The sequencing parameters established, were based on recommendations from WTCHG and those published by Conesa *et al*.^18^.

### Quasi-mapping

Transcripts were quantified via the Salmon (version 0.8.2) package using the transcriptome-based quasi-mapping mode^19^. The following commands were used during quasi-mapping in accordance with the Salmon guidelines: salmon quant –I transcripts_index -l ISR a −1/SampleX_lane1_mate1.fastq.gz/SampleX_lane2_mate1.fastq.gz −2/SampleX_lane1_mate2.fastq.gz/SampleX_lane2_mate2.fastq.gz -o Sample1–dumpEq–posBias–gcBias –writeUnmappedNames. The transcript index used during quasi-mapping was derived from the UCSC refseq rn6.0 mRNA library available at the following link: http://hgdownload.soe.ucsc.edu/goldenPath/rn6/bigZips/refMrna.fa.gz.

Data files were assigned alternative names to blind the experimenter during the relevant stages of the quasi-mapping analysis.

### Differential Expression Analysis

Once each sample was quantified, the data were imported into R and summarised to a gene-level using the ‘Tximport’ function (v1.4.0)^74^. Gene level differential expression analysis between Wistar and SHR samples was performed using the 'DESeq2' command in DESeq2 (version 1.16.1)^20^. Significance for differential expression was accepted at the Benjamini-Hochberg adjusted p < 0.05 level. The ‘LFCshrink’ function was used to shrink log fold changes after analysis as per the DESeq2 vignette^20^.

### Gene ontology and KEGG analysis

Gene ontology (GO) and KEGG over-representation tests were performed using ClusterProfiler (Version 3.4.4) with ‘EnrichGO’ and ‘EnrichKEGG’ functions respectively^22^. The differentially expressed gene input was taken at the Benjamini-Hochberg adjusted p < 0.05 level. Significant groups were subsequently determined using Benjamini-Hochberg correction and cut-off levels of *p* < 0.01 and *q* < 0.05 were enforced. Redundancy amongst GO terms was removed using the ClusterProfiler simplify command, where the adjusted *p-*value was used as the deciding variable, with a cut-off value of 0.7. The ClusterProfiler ‘Enrichmap’ function was used to generate KEGG enrichment maps^23–25^. We subsequently generated an integrated KEGG enrichment diagram by combining signalling pathways from the over-represented KEGG pathways ‘Circadian entrainment’, ‘Dopaminergic synapse’ and ‘Glutamatergic synapse’.

### cDNA Library Preparation for qRT-PCR

We used 50 ng stellate RNA for constructing the *q*RT-PCR cDNA libraries. For rat PGSN RNA, the SuperScript^TM^ III VILO^TM^ cDNA synthesis protocol was followed according to manufacturer’s instructions. Briefly, rat RNA samples (50 ng) were added to the III VILO reaction mix, SuperScript^TM^ enzyme mix and nuclease-free H_2_O. The samples were vortexed and incubated in a thermocycler (GStorm) at 25 °C (10 mins), 42 °C (60 mins), 80 °C (5 mins). For human PGSN RNA, the SuperScript^TM^ IV VILO^TM^ cDNA synthesis protocol was followed according to manufacturer’s instructions (ThermoFisher). Briefly, human RNA samples (50 ng) were added to ezDNAse buffer, exDNAse enzyme and nuclease-free H_2_O. The samples were mixed and incubated (37 °C, 2 min). SuperScript^TM^ IV VILO^TM^ master mix (or for control experiments SuperScript^TM^ IV VILO^TM^ no reverse transcriptase) was added to each sample with nuclease-free H_2_O. The samples were mixed and incubated in a thermocycler (GStorm, GS4822) at 25 °C (10 mins), 50 °C (10 mins), 85 °C (5 mins). Concentrations of cDNA in each sample and the 260/280 ratios were calculated (NanoDrop Lite) to detect the presence of contaminants. Samples with an abnormal 260/280 ratio (<1.7 and >1.95) were discarded. Samples were aliquoted and frozen at −80 °C for long-term storage or kept at 4 °C for immediate use.

### Two-Step Quantitative Real-Time PCR

*qRT*-PCR was used to confirm the presence of the following mRNA transcripts in the PGSN cDNA libraries: *Chrnb4* (Rn00583822_m1, Hs00609523_m1); *Gabra1* (Rn00788315_M1, Hs00971228_m1); *Gabra2* (Rn01413643_m1, Hs00168069_m1); *Glrb* (Rn00583966_M1, Hs00923871_m1); *Gria1* (Rn00709588_m1, Hs00181348_m1); *Gria2* (Rn00568514_M1 Hs00181331_m1); *Gria3* (Rn00583547_m1, Hs01557466_m1); *Grik1* (Rn01458414_M1, Hs00543710_m1); *Grik2* (Rn00570853_m1, Hs00222637_m1); *Grin2b* (Rn00680474_M1, Hs01002012_m1); *Htr3a* (Rn00667026_m1, Hs00168375_m1); *Htr3b* (Rn00573408_M1, Hs01573423_m1); *P2rx3* (Rn00579301_m1, Hs01125554_m1); *P2rx4* (Rn00580949_m1, Hs00902156_g1); *P2rx6* (Rn00562354_m1, Hs01003997_m1). The following controls were selected: beta-2-microglobulin (*B2m;* Rn00560865_m1, Hs00187842_m1; rat, human), glyceraldehyde-3-phosphate dehydrogenase (*Gapdh;* Rn99999916_s1, Hs02786624_g1; rat, human). TaqMan^®^ Gene Expression Master Mix (ThermoFisher) was added to each cDNA sample in addition to the selected primer conjugated to a FAM dye and nuclease-free H_2_O. cDNA samples or no reverse transcriptase controls were diluted 1:20 for optimal PCR reactions. A 96-well plate with 20 μL samples (3 replicates) were run on a real-time quantitative PCR thermocycler (ABI, PRISM). Temperatures were held at 50 °C (2 min) and 95 °C (10 min) before thermal cycling (40 cycles) under the following conditions 95 °C (15 s), 60 °C (1 min). Data displayed in the results section depicts gene counts normalised to *B2m;* however, each primer was analysed against two housekeeping genes, *B2m* and *Gapdh* for validation and accuracy of *q*RT-PCR data. The relative amount of each transcript was calculated using the comparative (C_T_) method (∆∆ C_T_, rat; ∆ C_T_, human)^75^.

### Statistical Analysis

*q*RT-PCR data were analysed using GraphPad Prism software (v7). When the data passed normality tests, an unpaired Student’s *t-*Test was used. When the data were not normally distributed, nonparametric tests were used with the specific statistical test reported in the figure legend. All *q*RT-PCR data are expressed as Log_2_ fold change ± SEM. Statistical significance was accepted at *p* < 0.05, unless otherwise described. For details of quality control and further methodology, please refer to^76^. 

### Data availability

Our RNA sequencing (RNAseq) raw FastQ files are deposited in the NCBI short reads archive under SRA number SRP132271 (NCBI Short Read Archive) and our quasi-mapped data will be available under Gene Expression Omnibus (GEO) accession number (GSE110197).

## Electronic supplementary material

Supplementary Dataset
