# Supplementary material for: RNA Sequencing Reveals Novel Transcripts from Sympathetic Stellate Ganglia During Cardiac Sympathetic Hyperactivity
Source: Sci Rep. 2018 Jun 5;8:8633. doi: 10.1038/s41598-018-26651-7 (PMC5988725; doi:10.1038/s41598-018-26651-7)
Supplement: Supplementary file 1 — Supplementary Dataset [file 41598_2018_26651_MOESM1_ESM.pdf]

RNA Sequencing Reveals Novel Transcripts from Sympathetic Stellate Ganglia During Cardiac Sympathetic Hyperactivity

*Emma N. Bardsley[1], Harvey Davis[1], Olujimi A. Ajijola[2], Keith J. Buckler[1], Jeffrey L. Ardell[2], Kalyanam Shivkumar[2], *David J. Paterson[1]

[1] Wellcome Trust OXION Initiative in Ion Channels and Disease, Burdon Sanderson Cardiac Science Centre, Department of Physiology, Anatomy and Genetics, Sherrington Building, University of Oxford, Oxford, OX1 3PT, UK
[2] UCLA Cardiac Arrhythmia Center, 100 Medical Plaza, Suite 660, Los Angeles, CA, 90095, USA

*Corresponding Authors: emma.bardsley@dpag.ox.ac.uk , david.paterson@dpag.ox.ac.uk

Funding: This project was funded by the Wellcome Trust OXION initiative (105409/Z/14/Z), the British Heart Foundation Centre of Research Excellence and BHF (RG/17/14/33085), NIH SPARC (OT2OD023848) and new innovator (DP2HL142045) initiatives.

Supplementary Figure 1

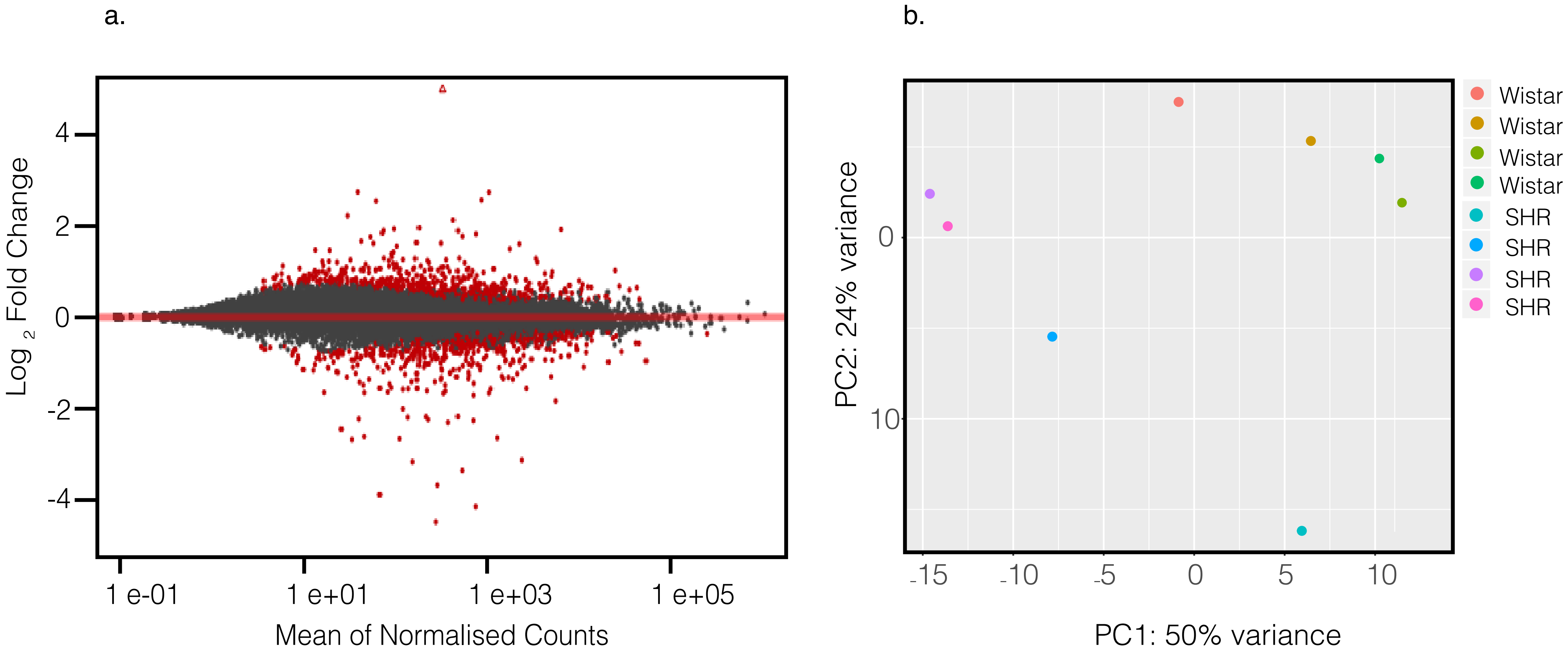

Supplementary figure 1 confirms the validity of the RNAseq analysis. The MA plot depicts the relationship between the number of counts per gene, the size of the fold change and the significance of fold changes. Significance is indicated by red spheres on a gene by gene basis. Non-significant transcripts are shown as grey spheres (a). A Principal Component (PCA) plot demonstrates the variation of the stellate ganglia transcriptome between Wistar and SHR strains (b).

Supplementary Table 1

Molecular GO ID	Molecular Function GO Description	Genes	Count	*p.* adjusted
GO:0005230	extracellular ligand-gated ion channel activity	*Chrnb4, Gabra1, Gabra2, Glrb, Gria1, Gria2, Gria3, Grik1, Grik2, Grin2b, Htr3a, Htr3b, P2rx3, P2rx4, P2rx6*	15	3.01E-05
GO:0042578	phosphoric ester hydrolase activity	*Acpp, Adora1, Ctdspl2, Dusp15, Enpp3, Fbp1, Gdpd3, Hmox1, Lpin2, Mtmr1, Napepld, Nt5dc1, Cyct, Pde2a, Pde6b, Pdxp, Pfkl, Pgap1, Phospho1, Plcxd2, Pld3, Ppm1f, Psph, Ptp4a1, Ptpdc1, Ptpn5, Ptpn7, Ptprn2, Ptpro, Sgpp2, Smpdl3b, Styxl1, Tmem55b*	33	1.72E-04
GO:0043177	organic acid binding	*Crabp2, Ddc, Egln3, Fabp3, Gclc, Glrb, Grik1, Grin2b, Grm7, Hba-a1, Id3, P3h1, Ptgds, S100a8, S100a9, Sele, Shmt2, Snca, Stx3, Tph1*	20	2.90E-03
GO:0008066	glutamate receptor activity	*Gria1, Gria2, Gria3, Grik1, Grik2, Grin2b, Grm7*	7	2.90E-03
GO:0022836	gated channel activity	*Cacna1c, Chrnb4, Clic3, Gabra1, Gabra2, Glrb, Gria1, Gria2, Gria3, Grik1, Grik2, Grin2b, Grm7, Hcn1, Htr3a, Htr3b, Kcnab2, Kcne5, Kcnh1, Kcnip4, Kcnj3, Oprm1, P2rx3, P2rx4, P2rx6, Piezo1, Scn2b*	27	3.59E-03
GO:0031406	carboxylic acid binding	*Crabp2, Ddc, Egln3, Fabp3, Gclc, Glrb, Grik1, Grin2b, Grm7, Id3, P3h1, Ptgds, S100a8, S100a9, Sele, Shmt2, Snca, Stx3, Tph1*	19	4.93E-03

Supplementary tablet 1 displays over-represented gene ontology (GO) groups invthe Molecular Function (MF) category. The over-represented GO groups and their respective genes are displayed after simplifying semantically redundant terms. The number of significant genes (count) and Benjamini-Hochburg *p*.adjusted (*p*.adj<0.01) values are listed. There were no over-represented Biological Processes GO groups between strains at the Benjamini-Hochburg *p*.adj<0.01.

Supplementary Table 2

Cellular Component GO ID	Cellular Component GO Description	Genes	Count	*p.* adjusted
GO:0033267	axon part	*Adcyap1, Adora1, Bloc1s6, Calb1, Calca, Camk2d, Cobl, Gria1, Gria2, Gria3, Grik1, Grik2, Grin2b, Grm7, Kcnab2, Kcnh1, Kif5b, P2rx3, P2rx4, Pfn2, Ptprn2, Sirt2, Slc18a3, Snca, Spg7, Spock1, Stx3, Syngr1, Tpx2*	29	8.11E-05
GO:0098793	presynapse	*Adcyap1, Adora1, Apba2, Cacna1c, Calb1, Calca, Ccl2, Ddc, Dixdc1, Gabra2, Gria1, Gria2, Gria3, Grik1, Grik2, Grin2b, Grm7, Hcn1, Kcnab2, Kcnh1, Lin7c, P2rx3, P2rx4, Pde2a, Pfn2, Ptprn2, Rab15, Rab8b, Rnf40, Slc18a1, Slc18a3, Snca, Stx3, Syngr1, Synpr*	35	1.13E-03
GO:0000323	lytic vacuole	*Acpp, Ahnak, Anxa2, Atp6v1b2, Atp6v1c1, Cd34, Chga, Cst3, Ctsz, Cxcr4, Cyb561a3, Cybrd1, Daglb, Dpp4, Fgfr3, Gaa, Glb1, Gm2a, Gnai3, Lmbrd1, Mt1, Ncoa4, P2rx4, Pik3c3, Rab27a, Slc11a1, Snx14, Snx2, Spata31a5, Stx3, Sult1c2, Sult1c2a, Tmem150c, Tmem55b, Tspan1, Vps26a, Zc3hav1*	37	1.21E-03
GO:0005764	lysosome	*Acpp, Ahnak, Anxa2, Atp6v1b2, Atp6v1c1, Cd34, Chga, Cst3, Ctsz, Cxcr4, Cyb561a3, Cybrd1, Daglb, Dpp4, Fgfr3, Gaa, Glb1, Gm2a, Gnai3, Lmbrd1, Mt1, Ncoa4, P2rx4, Pik3c3, Rab27a, Slc11a1, Snx14, Snx2, Spata31a5, Stx3, Sult1c2, Sult1c2a, Tmem150c, Tmem55b, Tspan1, Vps26a, Zc3hav1*	37	1.21E-03
GO:0045211	postsynaptic membrane	*Adora1, Cacna1c, Chrnb4, Cnih2, Dnaja3, Gabra1, Gabra2, Glrb, Gria1, Gria2, Gria3, Grik1, Grik2, Grin2b, Grm7, Htr3a, Htr3b, Kcnh1, Lin7c, Lrrtm3, Rgs14*	21	4.08E-03

Supplementary table 2 displays over-represented gene ontology (GO) groups in the Cellular Component (CC) category. The over-represented GO groups and their respective genes are displayed after simplifying semantically redundant terms. The number of significant genes (count) and Benjamini-Hochburg *p*.adjusted (*p*.adj<0.01) values are listed. There were no over-represented Biological Processes GO groups between strains at the Benjamini-Hochburg *p*.adj<0.01.

Supplementary Table 3

KEGG Functional Enrichment ID	KEGG Functional Enrichment Description	Genes	Count	*p.* adjusted
rno04713	Circadian entrainment	*Gnai3, Gria3, Prkg2, Kcnj3, Gria2, Grin2b, Gria1, Camk2d, Gucy1a3, Gnb3, Prkacb, Cacna1c, Adcy10, Gng7*	14	4.24E-03
rno04728	Dopaminergic synapse	*Ddc, Gnai3, Kif5b, Kif5a, Gria3, Kcnj3, Gria2, Grin2b, Gria1, Mapk14, Camk2d, Prkacb, Gnb3, Slc18a1, Cacna1c, Gng7*	16	4.24E-03
rno04723	Retrograde endocannabinoid signaling	*Cacna1c, Daglb, Gabra1, Gabra2, Gnai3, Gnb3, Gng7, Gria1, Gria2, Gria3, Kcnj3, Mapk14, Napepld, Ndufa4l2, Ndufv3, Prkacb*	16	1.96E-02
rno04724	Glutamatergic synapse	*Gnai3, Grik1, Grik2, Gria3, Kcnj3, Gria2, Grin2b, Gria1, Grm7, Gnb3, Prkacb, Cacna1c, Gng7*	13	2.59E-02
rno04080	Neuroactive ligand-receptor interaction	*Adora1, Adrb2, Agtr2, Bdkrb2, Chrnb4, F2rl2, Gabra1, Gabra2, Glrb1, Gpr35, Gria1, Gria2, Gria3, Grik1, Grik2, Grin2b, Grm7, Hcrtr1, Oprm1, P2rx3, P2rx4, P2rx6, Ptger2*	23	2.86E-02
rno05032	Morphine addiction	*Adora1, Gabra1, Gabra2, Gnai3, Gnb3, Gng7, Kcnj3, Oprm1, Pde11a, Pde2a, Prkacb*	11	2.86E-02

Supplementary table 3 depicts the over-represented KEGG functional enrichment pathways. The over-represented KEGG pathways and the respective genes are displayed after simplifying semantically redundant terms. The number of significant genes (count) and the Benjamini-Hochburg *p*.adjusted (*p*. adj<0.01) values are listed. Groups that were identified with <10 genes are excluded from this dataset.
